# Supplementary material for: Circulating HHIP Levels in Women with Insulin Resistance and PCOS: Effects of Physical Activity, Cold Stimulation and Anti-Diabetic Drug Therapy
Source: J Clin Med. 2023 Jan 22;12(3):888. doi: 10.3390/jcm12030888 (PMC9918013; doi:10.3390/jcm12030888)
Supplement: Supplementary file 1 [file jcm-12-00888-s001.zip › jcm-2147137-supplementary.pdf]

Table S1. Row mean scores differ and Cochran-Armitage trend analysis

	IR		PCOS	
**Model adjusted**	**$\chi2$**	**P-value**	**$\chi2$**	**P-value**
Row Mean Scores Test	126.8853	0.0001	85.3524	0.0001
Cochran-Armitage Trend Test	-11.0927	0.0001	-11.7486	0.0001

PCOS, polycystic ovary syndrome; IR, Insulin resistance; HHIP levels were cut-off, and adjusted for age, BMI, WHR, SBP, DBP and lipid profile.

Table S2. Main clinical and metabolic features pre-and post-treatment with anti-diabetes drug in PCOS women

	**Metformin (n = 45)**		**GLP-1RA (n = 52)**		**TZDs (n = 40)**	
	**Pre-treatment**	**Post-treatment**	**Pre-treatment**	**Post-treatment**	**Pre-treatment**	**Post-treatment**
BMI (kg/m^2)	24.4(22.4-26.9)	23.1(21.4-25.7)*	27.9(25.7-30.9)	25.5(23.6-28.6)**	23.4(21.8-25.9)	22.9(20.6-25.5)
WHR	0.86(0.80-0.89)	0.82(0.78-0.88)**	0.89(0.85-0.92)	0.86(0.81-0.89)**	0.86(0.80-0.90)	0.81(0.77-0.87)**
SBP (mmHg)	112(104-120)	116(110-120)	118(111-123)	108(104-120)**	116(107-121)	112(105-119)
DBP (mmHg)	72(68-80)	76(67-80)	72(67-80)	72(66-78)	69(64-77)	70(65-78)
TG (mmol/L)	1.30(1.06-1.87)	1.23(0.92-1.62)	1.94(1.50-2.50)	1.15(0.81-1.79)**	1.47(1.23-1.82)	1.18(1.01-1.68)
TC (mmol/L)	4.39(3.89-5.13)	4.18(3.79-4.69)**	4.71(4.18-5.35)	4.01(3.54-4.82)**	4.62(3.98-5.08)	4.15(3.65-4.65)**
HDL-C(mmol/L)	1.19(1.04-1.35)	1.30(1.13-1.52)	1.09(0.98-1.26)	1.11(0.98-1.29)	1.17(1.03-1.35)	1.32(1.17-1.56)**
LDL-C (mmol/L)	2.50(1.96-3.14)	2.10(1.82-2.66)**	2.74(2.20-3.28)	2.18(1.95-2.70)**	2.49(2.11-3.01)	2.25(1.76-2.59)**
FFA (μmol/L)	0.53(0.44-0.68)	0.45(0.35-0.55)*	0.46(0.35-0.57)	0.38(0.27-0.45)**	0.52(0.39-0.79)	0.40(0.26-0.57)**
FBG (mmol/L)	5.11(4.75-5.57)	5.07(4.62-5.29)*	5.38(5.16-5.78)	5.04(4.71-5.23)**	5.17(4.89-5.48)	4.94(4.75-5.35)**
2h-BG (mmol/L)	7.88(6.40-9.06)	6.34(5.71-7.17)**	8.29(7.28-10.10)	6.66(5.62-8.39)**	7.77(6.66-8.96)	6.79(5.77-8.37)**
FIns (mU/L)	17.44(13.44-23.37)	13.30(9.01-16.8)**	27.33(17.95-39.55)	15.83(10.23-22.38)**	14.00(10.34-19.50)	10.64(6.73-13.55)**
2h-Ins (mU/L)	129.5(93.2-208.3)	96.2(56.8-133.9)**	176.4(128.9-337.2)	108.3(73.1-173.4)**	138.3(99.2-229.8)	84.9(58.9-138.8)**
HbA1c (%)	5.5(5.2-5.6)	5.3(5.0-5.5)**	5.6(5.3-5.9)	5.2(5.0-5.4)**	5.4(5.2-5.7)	5.3(5.05-5.5)**
AUC_g	15.9(14.1-19.0)	15.0(13.8-17.6)*	17.6(16.2-20.3)	14.9(13.3-17.3)**	17.2(15.7-18.9)	15.0(13.4-17.0)**
AUC_i	270.2(168.2-330.1)	198.7(136.5-248.8)**	351.7(236.5-519.0)	274.1(183.3-391.0)**	269.3(196.2-384.5)	160.7(111.6-231.2)**
M-value (mg/kg/min)	4.77(4.06-6.02)	6.28(4.75-7.50)**	3.32(2.86-3.78)	4.43(3.63-6.20)**	5.21(4.08-5.97)	5.51(4.91-7.02)**
HOMA-IR	4.08(2.72-5.41)	2.86(1.88-4.06)**	6.54(4.41-9.78)	3.41(2.13-5.03)**	3.14(2.27-4.72)	2.33(1.52-3.13)**
VAI	2.09(1.53-2.94)	1.69(1.22-2.36)	3.22(2.05-4.64)	1.94(1.18-3.25)**	2.12(1.70-2.96)	1.68(1.11-2.53)*
BAI	29.8(26.9-31.8)	28.88(26.68-31.34)	32.8(30.3-35.4)	30.9(28.9-33.1)**	28.4(27.2-31.0)	28.2(26.7-31.4)

PCOS, polycystic ovary syndrome; IR, Insulin resistance; BMI, body mass index; WHR, waist-to-hip ratio; SBP, systolic blood pressure; DBP, diastolic blood pressure; TG, triglyceride; TC, total cholesterol; HDL-C, high-density lipoprotein cholesterol; LDL-C, low-density lipoprotein cholesterol; FFA, free fatty acids; FBG, fasting blood glucose; 2h-PBG, 2h-post-glucose load blood glucose; FIns, fasting plasma insulin; 2h-Ins, 2h-plasma insulin after glucose overload; AUC_g, the area under the curve for glucose; AUC_i, the area under the curve for insulin; HOMA-IR, HOMA-insulin resistance index; VAI, visceral adiposity index; BAI, body adiposity index; Values are given as median (Interquartile Range).*p <0.05, **p < 0.01 compared with baseline.

Table S3. Hormone and cytokine levels pre-and post-treatment with anti-diabetes drug in PCOS women

	Metformin (n = 45)		GLP-1RA (n = 52)		TZDs (n = 40)	
	**Pre-treatment**	**Post-treatment**	**Pre-treatment**	**Post-treatment**	**Pre-treatment**	**Post-treatment**
DHEA-S (μg/dL)	220.3(164.7-282.6)	194.2(157.7-223.7)**	247.2(164.4-361.8)	274.0(140.1-360.7)	251.1(172.8-315.2)	243.8(174.3-301.0)
E2 (pg/mL)	49.0(31.7-64.8)	52.5(32.6-66.2)	41.1(31.7-50.6)	42.2(30.1-85.7)*	36.9(26.4-50.7)	52.2(37.5-110.5)*
LH (IU/L)	6.50(3.61-11.53)	4.79(3.47-8.50)	7.06(4.79-10.13)	3.73(2.17-7.86)**	8.03(3.89-11.78)	5.47(3.28-7.60)
FSH (IU/L)	7.20(5.72-8.97)	7.34(5.89-8.40)	5.82(4.73-6.73)	5.87(4.09-7.53)	6.00(5.52-6.62)	6.21(5.07-7.44)
Prog (ng/mL)	1.84(1.20-2.44)	1.90(1.39-2.95)	1.49(0.89-2.27)	1.44(0.96-3.16)*	1.57(1.20-2.46)	1.88(1.43-2.75)
SHBG (nmol/L)	32.1(22.3-43.2)	45.6(32.0-84.0)**	30.0(21.7-43.1)	37.2(20.5-55.3)	34.9(26.7-49.8)	54.1(42.0-74.9)**
TEST (nmol/L)	2.30(1.83-2.92)	1.99(1.39-2.58)	1.94(1.31-2.22)	1.30(0.97-1.86)**	2.15(1.39-2.65)	1.55(1.14-1.80)**
FAI	7.01(4.22-10.19)	3.75(2.47-6.67)**	6.27(3.59-9.12)	3.31(2.02-6.46)**	5.71(3.81-8.72)	2.72(1.80-4.24)**
Adipoq (mg/L)	29.8(23.8-35.2)	38.5(29.1-47.4)**	25.9(22.4-33.2)	39.0(30.1-48.3)**	23.5(20.2-31.5)	37.2(27.9-44.6)**
HHIP(μg/L)	14.7(11.4-17.3)	14.9(12.3-17.3)	15.3(11.9-18.3)	12.2(10.2-14.8)**	15.7(12.4-17.8)	13.0(10.4-16.4)*

DHEA-S: Dehydroepiandrosterone sulfate; E2, estradiol; LH, Luteinizing hormone; FSH, Follicle- stimulating hormone; Prog, Progesterone; SHBG, Sex-hormone binding globulin; TEST, testosterone; FAI, free androgen index = T(nmol/l)/SHBG (nmol/l) × 100. Adipoq, adiponectin. Values are given as median (Interquartile Range). *p <0.05, **p < 0.01 compared with baseline.
